# Prognostic role of different PD-L1 expression patterns and tumor-infiltrating lymphocytes in high-grade serous ovarian cancer: a systematic review and meta-analysis

**DOI:** 10.3389/fimmu.2023.1234894

**Published:** 2023-08-15

**Authors:** Ye-Min Wang, Wei Cai, Qing-Ming Xue, Jin-Yao Zhang, Lv Zhou, Su-Yi Xiong, Huan Deng

**Affiliations:** ^1^ Department of Pathology, the Fourth Affiliated Hospital of Nanchang University, Nanchang, Jiangxi, China; ^2^ Medical College, Nanchang University, Nanchang, Jiangxi, China

**Keywords:** high-grade serous ovarian cancer, PD-L1, tumor-infiltrating lymphocytes, prognosis, meta-analysis

## Abstract

**Background:**

The prognostic value of programmed cell death ligand 1 (PD-L1) expression and tumor-infiltrating lymphocytes (TILs) in high-grade serous ovarian cancer (HGSOC) remains a controversial topic in the research field. To comprehensively assess the importance of PD-L1 and TILs in this particular subtype of ovarian cancer, we performed a meta-analysis.

**Methods:**

We conducted a comprehensive search of PubMed, Embase, Scopus, Web of Science, and Cochrane Library databases up to December 25, 2022. The association between PD-L1, TILs, and survival outcomes was evaluated using the combined hazard ratios (HRs) and their corresponding 95% confidence intervals (CIs).

**Results:**

This meta-analysis comprised 11 trials involving a total of 1746 cases. The results revealed no significant association between PD-L1 expression in tumor cells (TCs) and overall survival (OS, HR = 0.76, 95% CI: 0.52-1.09, *p* = 0.136) or progression-free survival (PFS, HR = 0.71, 95% CI: 0.4 -1.24, *p* = 0.230). Nevertheless, a correlation was observed between PD-L1 expression in immune cells (ICs) and OS (HR = 0.73, 95% CI: 0.55-0.97, *p* = 0.031). Furthermore, the presence of CD8^+^ and PD-1^+^ TILs was found to significantly enhance OS (HR = 0.70, 95% CI = 0.55-0.87, *p* = 0.002; HR = 0.57, 95% CI = 0.40-0.80, *p* = 0.001, respectively) and PFS (HR = 0.62, 95% CI = 0.41-0.92, *p* = 0.019; HR = 0.52, 95% CI = 0.35-0.78, *p* = 0.002, respectively), whereas the presence of CD3^+^ and CD4^+^ TILs was positively associated with OS (HR = 0.50, 95% CI = 0.29-0.87, *p* = 0.014; HR = 0.55, 95% CI = 0.34-0.91, *p* = 0.020, respectively).

**Conclusion:**

This study indicates a positive correlation between ICs-derived PD-L1 and survival, while no significant correlation was observed between TCs-derived PD-L1 and prognosis. These results highlight the importance of studying PD-L1 expression in ICs as a prognostic predictor. In addition, the presence of TILs was found to significantly improve patient survival, suggesting that TILs may be a valuable prognostic biomarker.

**Systematic review registration:**

https://www.crd.york.ac.uk/prospero/, identifier CRD42022366411.

## 1 Introduction

High-grade serous ovarian cancer (HGSOC) accounts for up to 85% of ovarian cancer and represents the most aggressive histologic type (1). Due to the lack of specific signs and reliable screening tools, this tumor is often diagnosed at an advanced stage (FIGO stage III-IV) with an unacceptable five-year survival rate. Despite surgery and platinum-based chemotherapy regimens, more than 70% of patients develop recurrence, metastasis, and drug resistance (2). The establishment of prognostic factors is crucial in determining the clinical management and treatment strategies for HGSOC. It has been proposed that chemotherapy sensitivity, tumor stage, and cytoreductive surgery are associated with the prognosis of HGSOC (3), but these factors still lack considerable accuracy and specificity in predicting the survival of individual patients. Therefore, there is an urgent need to unveil more novel and reliable biomarkers to guide the tailored treatment and predict survival outcomes of HGSOC patients.

**Figure 1:**
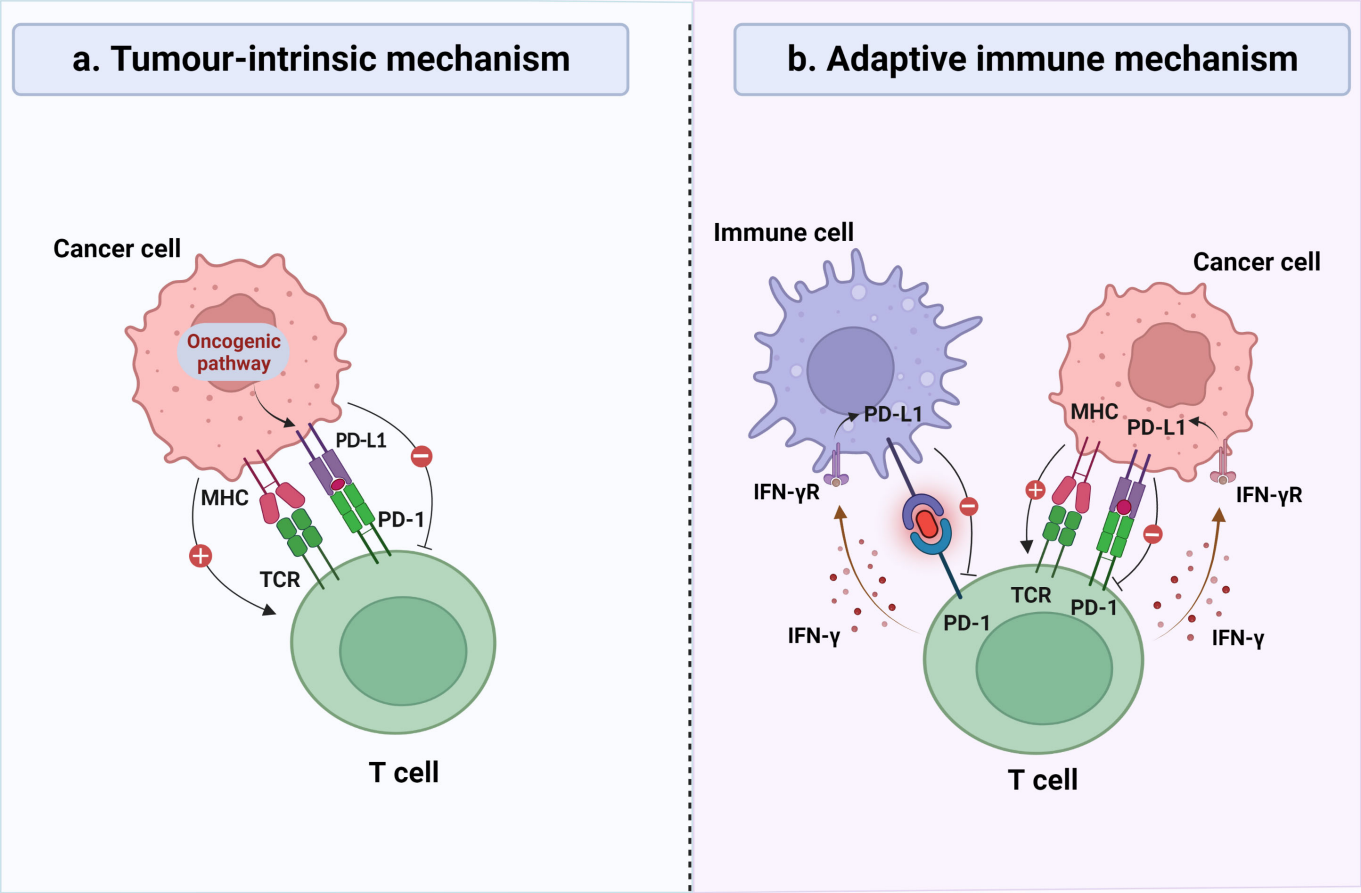
General mechanisms of PD-L1 expression on tumor cells (TCs) and immune cells (ICs). **(A)** In certain types of cancer, continuous activation of the oncogenic signaling cascades may lead to overexpression of PD-L1 on the surface of TCs, thereby inhibiting the recognition and elimination of TCs by cytotoxic T lymphocytes, regardless of the inflammatory signaling status in the tumor microenvironment. **(B)** In certain types of cancer, the expression of PD-L1 was observed on both TCs and ICs, which is induced by inflammatory signaling generated in response to a robust antitumor immune response, representing an adaptive immune response mechanism. IFN-γ, interferon-γ; MHC, major histocompatibility complex; TCR, T-cell receptor.

Cancer research has been long hampered by the variation of intrinsic tumor factors. With the development of genomic and molecular techniques, accumulating evidence provides more insights into the central role of the tumor microenvironment (TME). Tumor-infiltrating lymphocytes (TILs), an important component of the TME, exert bidirectional effects on the antitumor immune response. TILs represent a heterogeneous population of T cells, generally localized in the tumor stroma or epithelium, and are capable of recognizing tumor antigens and killing malignant cells (4, 5). Unfortunately, cancer cells can skillfully utilize multiple pathways to create an immunosuppressive microenvironment and evade anti-tumor immune responses. The programmed death ligand 1 (PD-L1)/programmed cell death-1 (PD-1) axis is considered a crucial pathway for this. PD-1, belonging to the CD28 receptor family, is prominently found in activated TILs and possesses the capability to impede T cell proliferation and cytokine secretion, thereby culminating in T cell exhaustion (6). PD-L1, the main ligand of PD-1, belongs to the B7 family and is mainly expressed in tumor cells (TCs) and immune cells (ICs), but the regulatory mechanisms of the two are different (**Figure 1**) (7). In TCs, PD-L1 expression is primarily governed by tumor intrinsic mechanisms (8–10), while in ICs, it depends on an adaptive immune mechanism (11). The upregulation of PD-L1 leads to its specific binding to PD-1 on the surface of T cells, thereby inhibiting the function of local effector T cells and enabling TCs to successfully evade immune surveillance (12, 13). In recent years, PD-L1 blockade therapies have shown unprecedented efficacy against a variety of tumors. There is growing evidence that TILs and PD-L1 are strongly associated with the prognosis of cancer patients, including breast cancer, non-small cell lung cancer, colorectal cancer, and melanoma (14–17). However, studies on the prognostic impact of TILs and PD-L1 on HGSOC are scarce and often contradictory (18–20).

Our objective was to evaluate the potential prognostic significance of PD-L1 and TILs in HGSOC by conducting a thorough analysis of all scientific studies that met our eligibility criteria. It is worth highlighting that recent research indicates that the expression of PD-L1 in ICs is directly associated with the response of patients with solid tumors to PD-L1 targeting antibody MPDL3280A, whereas such a correlation is not observed in TCs (21). Consequently, we examined the prognostic value of PD-L1 in two distinct expression patterns, specifically TCs and ICs. In contrast to previous meta-analyses (22, 23), our focus was solely on HGSOC, as it exhibits a more prevalent immune infiltration phenomenon and distinctive immunological features when compared to other histological subtypes (24). In addition, the interactions between PD-L1, TILs, and survival appears to be found only in HGSOC (25), suggesting the possibility that patients with this subtype may benefit more from immunotherapy. To our knowledge, this is the first meta-analysis to comprehensively explore the relationship between TILs, PD-L1, and prognosis in patients with HGSOC.

## 2 Materials and methods

### 2.1 Protocol and eligibility criteria

This systematic review and meta-analysis has been registered in PROSPERO (CRD42022366411) and its reporting adheres to the guidelines outlined in the Preferred Reporting Items for Systematic Reviews and Meta-Analyses (PRISMA) 2020 statement (26) (**Supplementary Table 1**).

The selection of studies was based on the following criteria: (1) patients were diagnosed with primary HGSOC by histopathology; (2) the immunohistochemistry technique was employed to determine the levels of PD-L1 expression and the density of TILs in HGSOC; (3) studies assessed the correlation among PD-L1, TILs, and survival outcomes, including overall survival (OS), progression-free survival (PFS), disease-free survival (DFS), and disease-specific survival (DSS); (4) articles were published in English. Conversely, we excluded studies that met the following requirements: (1) duplicate publications; (2) studies that had inadequate data to calculate Hazard Ratios (HRs) and 95% confidence intervals (CIs); (3) reviews, annals, conference abstracts, or letters.

### 2.2 Information sources and search criteria

We searched five databases: PubMed, Web of Science, Embase, the Cochrane Library and Scopus. There were no constraints on time, gender, or age. This review’s coverage dates began with the creation of each database and ended on December 25, 2022. The keywords were searched as follows: (“PD-L1” OR “B7-H1” OR “CD274” OR “programmed death ligand 1” OR “programmed cell death ligand 1” OR “PD L1 Protein” OR “B7-H1 antigen” OR “CD274 antigen”) AND (“Ovarian Neoplasms” OR “Ovarian Cancer” OR “Ovary Neoplasm” OR “Ovary Cancers” OR “Cancer of Ovary”) AND (“Tumor-Infiltrating Lymphocytes” OR “Tumor Infiltrating Lymphocyte” OR “Tumor-Derived Activated Cells” OR “Tumor Derived Activated Cells” OR “TILs”) (**Supplementary Table 2**). Moreover, we reviewed the reference lists of the studies ultimately included to identify potential studies.

### 2.3 Study selection

We imported the retrieved studies into EndNote (version X9) and removed duplicates. Two researchers (LZ and WC) independently reviewed the title and abstract of each study, after excluding unrelated studies, two more researchers (QMX and JYZ) independently reviewed the full text of the remaining studies according to the same criteria, and a third researcher (YMW) ruled on any differing points of view.

### 2.4 Data collection process and data items

We pre-prepared and tested a standardized form for data extraction. Two investigators (QMX and JYZ) independently extracted the first author’s name, year of publication, country, sample size, age, method of assessing PD-L1 and TILs, cut-off values, location of PD-L1 and TILs, TILs subtypes, median follow-up, and survival outcomes from each study. Any disagreements that arose were discussed and a consensus was reached.

### 2.5 Quality assessment

YMW and WC conducted a rigorous quality evaluation of each included study through the implementation of the Newcastle-Ottawa Scale (NOS) assessment tool (27). Specifically, the assessment focused on three critical dimensions, namely selection (0-4 points), comparability (0-2 points), and outcome (0-3 points), resulting in the assignment of a predetermined number of stars (≥6 stars were deemed high quality). If any disputes arise, a third reviewer, SYX, will be consulted to settle the matter.

### 2.6 Statistical analysis

We assessed the prognostic impacts of PD-L1 expression and TILs on HGSOC by pooling the HRs and 95% CIs of all included studies. The Cochrane Q test (χ^2^ test) and the I^2^ statistic were used to determine whether there was heterogeneity among studies. I^2^ > 50% and *p* < 0.05 were considered to be heterogeneous and the combined effect was calculated using a random-effects model (REM); otherwise, a fixed-effects model was used (FEM).

The publication bias was assessed using Egger’s linear regression test (28) and Begg’s funnel plot (29). The method of leave-one-out was employed to assess the sensitivity analysis of the results. We used STATA version 12.0 (Stata Corp LP, Texas, USA) for all the statistical analyses described above. The statistical significance level was set at a two-sided *p* value of 0.05.

## 3 Results

### 3.1 Literature search

**Figure 2:**
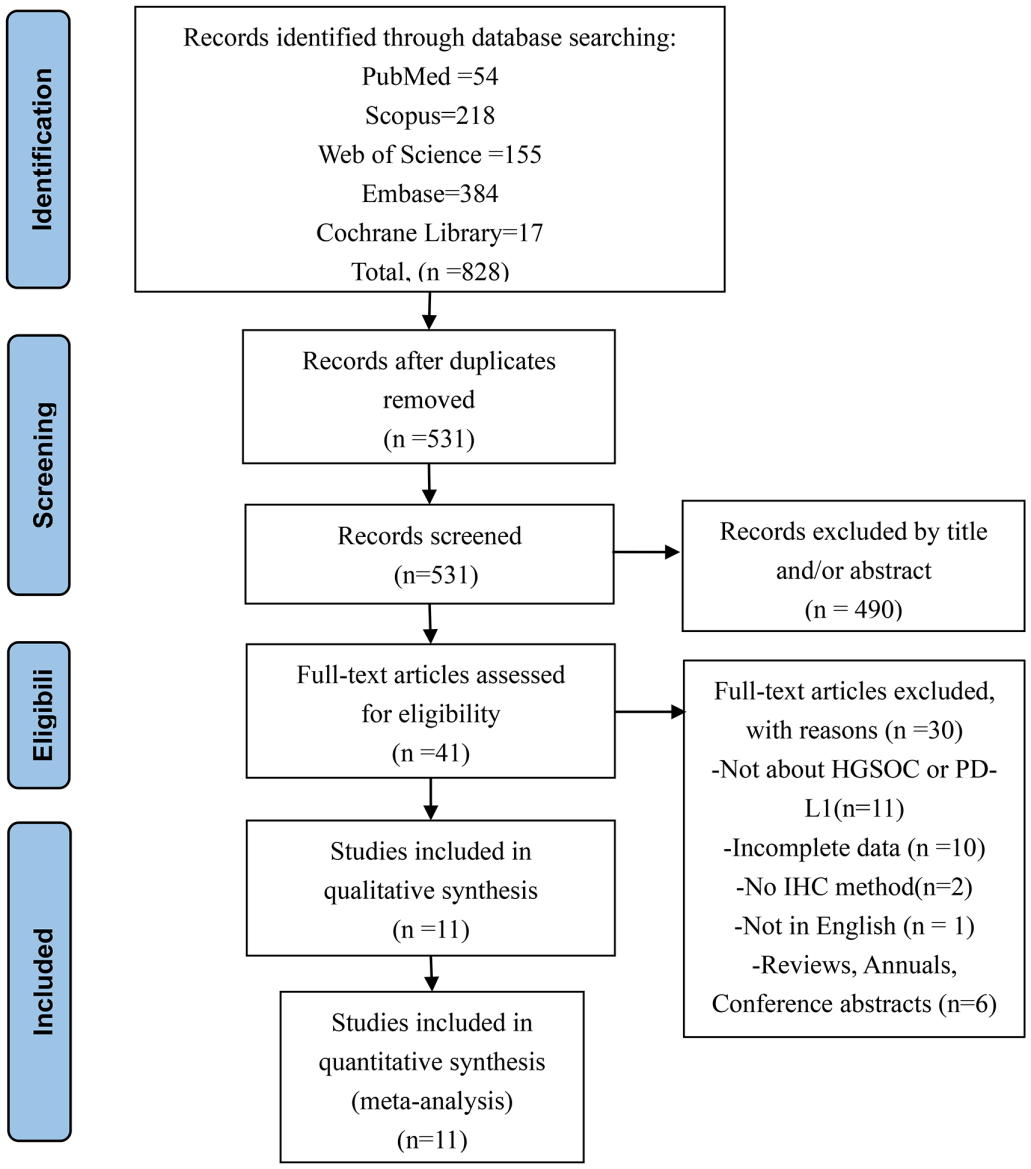
Flowchart of the study selection process.

Our team retrieved a total of 828 records from five different databases. Upon further examination, 297 duplicate records were excluded from our analysis. The remaining 531 studies were screened based on their titles and abstracts, and 490 were deemed irrelevant and removed from our investigation. After conducting a full analysis of the remaining 41 studies, 11 were deemed suitable for inclusion in our quantitative and qualitative analyses. A detailed overview of the search process, including the specific reasons for study exclusion, can be found in **Figure 2**.

### 3.2 Study characteristics

**Table 1** contains a comprehensive overview of the 11 studies that were included. Collectively, these studies encapsulate a patient cohort of 1746 individuals. The sample sizes from which these studies were conducted ranged from 100 to 283. The median age of the patients ranged from 50.8 to 67 years, and the median follow-up time extended from 30 to 156 months. There were four European studies, three Asian studies, three North American studies, and one South American study. Various studies employed immunohistochemical methodologies to evaluate PD-L1 and TILs, applying distinct cut-off values and scoring systems. Seven studies examined the correlation between TCs PD-L1 expression and associated prognoses, while five studies investigated the prognostic relevance of PD-L1 expression in ICs. Moreover, five studies concentrated solely on the relationship between intraepithelial TILs and survival. Finally, three studies probed the prognostic ramifications of intraepithelial and stromal TILs. According to NOS scores, all included studies scored ≥6, indicating the high methodological quality of these studies. Specific scoring details are shown in **Table 2**.

**Table 1:** Basic characteristics of included studies.

Author/Year	Country	Number of Patients	Age Median (range)	Detection method	Cut-Off value of PD-L1 Positive	Cut-Off value of TILs Positive	Location of PD-L1	Location of TILs	TILs subtype	Median follow-up (m)	Outcome	NOS score
Chen 2020	USA	100	53 (37-72)	IHC	TPS≥1%; CPS≥1	density for each marker	TC	IT	CD8	NR	OS, PFS	7
Darb-Esfahani 2016	Germany	215	60	IHC	IRS>0	density for each marker	TC	IT	PD-1, CD3	NR	OS, PFS	6
Henriksen 2020	Denmark	283	63	IHC	≥1%	≥80 cells/mm^2^	TC	IT	CD8	156	OS	8
Kim 2018	Korea	131	58 (32-79)	IHC	TC≥25%; IC≥5%	ST ≥50%; IT ≥10%	TC, IC	IT, ST	CD8, PD-1, FOXP3	30	OS, PFS	8
Martin de la Fuente 2020	Sweden	130	67 (43-86)	IHC	≥1%	≥1%	IC	IT	CD3, PD-1	39	OS	8
Mills 2019	US	112	59.7 (69.7-49.7)	IHC	≥1%	≥12.95 cells/HPF	TC, IC	IT	CD8, FOXP3	NR	OS	8
Pinto 2018	Chile	128	57 (29-83)	IHC	score≥2	≥10 cells/HPF	TC	IT, ST	CD3, CD4, CD8	NR	OS, PFS	7
Strickland 2016	US	245	NR	IHC	TC≥5%; IC≥1 cell/HPF	≥13 cells/HPF	TC, IC	IT	CD3, CD8, PD-1	NR	OS, DFS	6
Wang 2017	China	107	57 (29–82)	IHC	≥5%	score > 2 or 3	TC, IC	IT, ST	CD3, CD4, CD8	NR	OS	7
Webb 2016	Canada	195	NR	IHC	≥1 cell/HPF	≥5 cells/HPF	IC	IT	CD3, CD8, PD-1, FOXP3	NR	DSS	6
Bansal 2021	India	100	50.8 (31.5-82)	IHC	≥ 10%	score > 1	TC, IC	IT, ST	CD4, CD8	NR	OS, DFS	6

US, United States; NR, not reported; IHC, immunohistochemistry; HPF, high power field; CPS, combined positive score; TPS, tumor proportion score; IRS, immuno-reactivity score; TC, tumor cell; IC, immune cell; IT, intraepithelial; ST, stromal; OS, overall survival; PFS, progression-free survival; DFS, disease-free survival; DSS, disease-specific survival; NOS, Newcastle-Ottawa scale.

**Table 2:** The NOS quality assessment of the enrolled studies.

Study	Selection				Comparability	Outcome			Total Score
	Representativeness of exposed cohort	Selection of non-exposed cohort	Exposure ascertainment	Outcome not present prior to exposure	Control for factor	Assessment of outcome	Follow-up long enough	Adequacy of follow-up of cohorts	0-9
Chen 2020	⋆	⋆	⋆	⋆	⋆⋆	⋆	☆	☆	7
Darb-Esfahani 2016	⋆	⋆	☆	⋆	⋆⋆	⋆	☆	☆	6
Henriksen 2020	⋆	⋆	⋆	⋆	⋆⋆	⋆	⋆	☆	8
Martin de la Fuente 2020	⋆	⋆	☆	⋆	⋆⋆	⋆	⋆	⋆	8
Mills 2019	⋆	⋆	⋆	⋆	⋆⋆	⋆	⋆	☆	8
Pinto 2018	⋆	⋆	☆	⋆	⋆⋆	⋆	☆	⋆	7
Strickland 2016	⋆	⋆	☆	⋆	⋆⋆	☆	☆	⋆	6
Wang 2017	⋆	⋆	⋆	⋆	⋆⋆	⋆	☆	☆	7
Webb 2016	⋆	⋆	☆	⋆	⋆⋆	⋆	☆	☆	6
Bansal 2021	⋆	⋆	☆	⋆	⋆⋆	☆	☆	⋆	6
Kim 2018	⋆	⋆	☆	⋆	⋆⋆	⋆	⋆	⋆	8

NOS, Newcastle-Ottawa scale.

☆, zero score; ★, one score.

### 3.3 PD-L1 and prognosis

#### 3.3.1 TCs PD-L1 expression and prognosis

**Figure 3:**
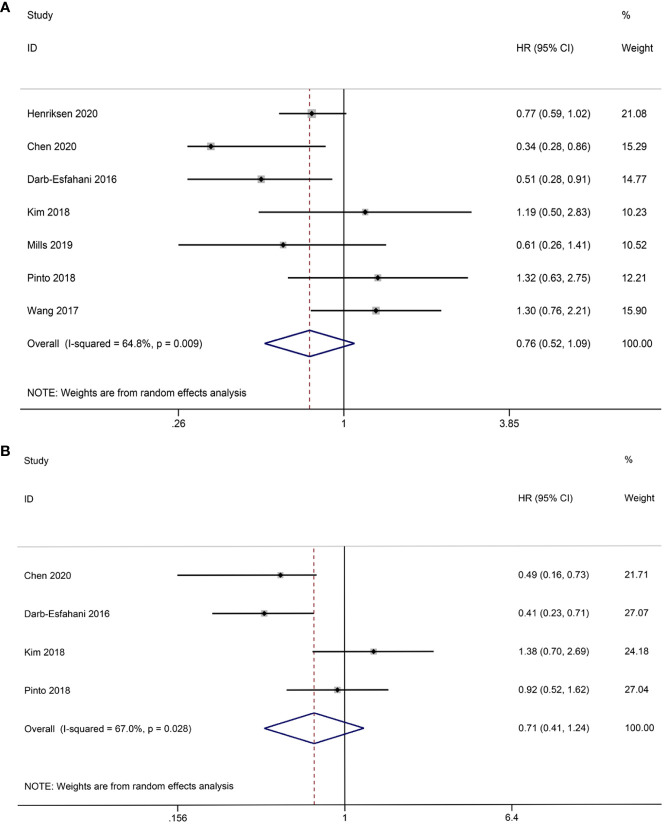
Forest plot of the relationship between TCs PD-L1 expression and survival outcomes in HGSOC. **(A)** Forest plots of overall survival (OS); **(B)** Forest plots of progression-free survival (PFS).

The association between TCs PD-L1 expression and OS has been investigated in seven studies. The

combined data analysis revealed that there was no significant correlation (HR = 0.76, 95% CI: 0.52-1.09, *p* = 0.136), which was further confirmed using REM due to the presence of heterogeneity (I^2 ^= 64.8%, *p* = 0.009) (**Figure 3A**). Additionally, data from four studies were analyzed to determine the relationship between TCs PD-L1 expression and PFS. Pooled results showed that TCs PD-L1 expression was not significantly correlated with prognosis (HR = 0.71, 95% CI: 0.41-1.24, *p* = 0.230). Given the slight heterogeneity among the four studies (I^2 ^= 67.0%, *p* = 0.028), REM was employed (**Figure 3B**).

#### 3.3.2 ICs PD-L1 expression and prognosis

**Figure 4:**
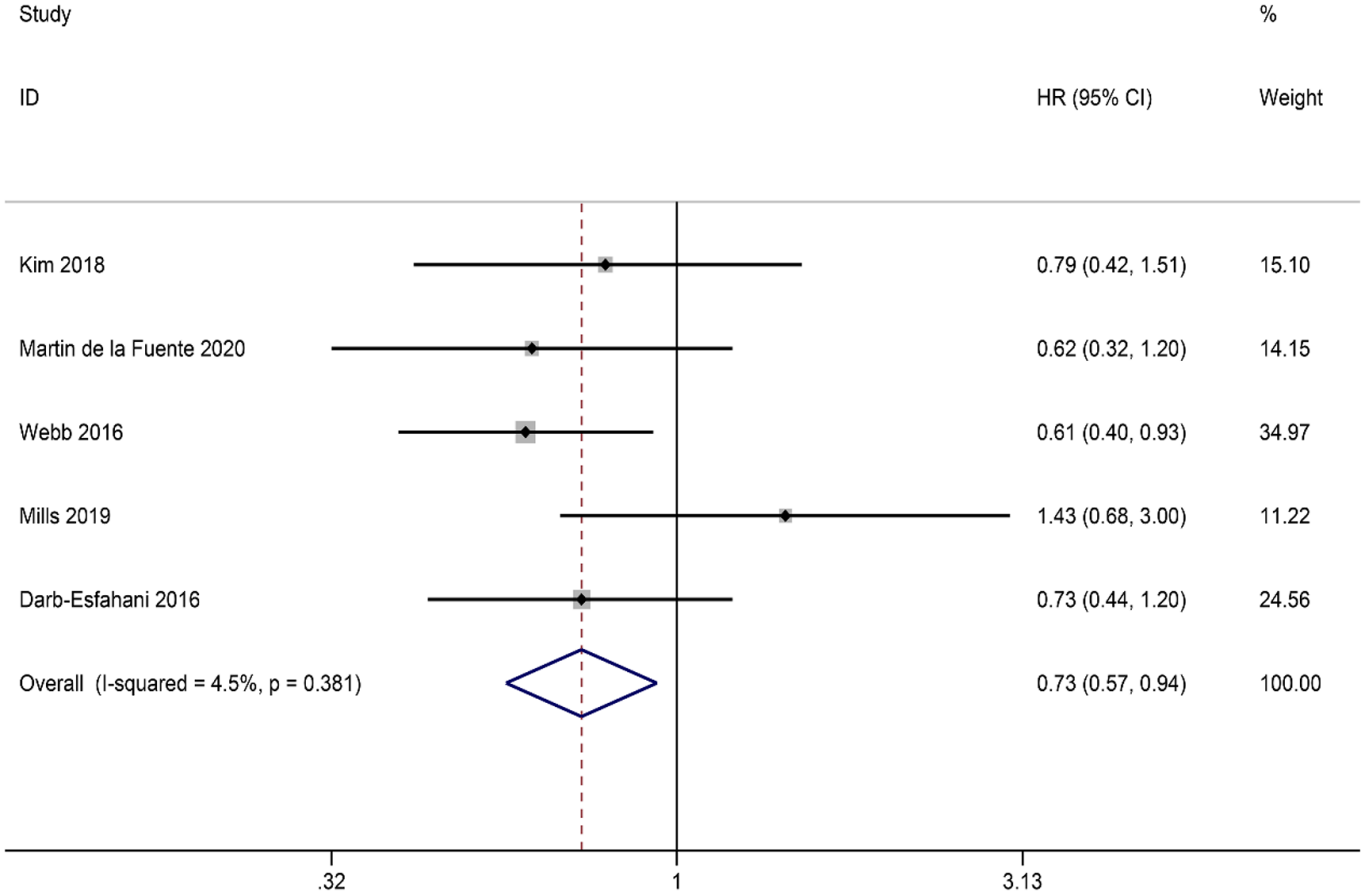
Forest plot of the relationship between ICs PD-L1 expression and overall survival (OS) in HGSOC.

Due to the limited availability of PFS data, our study specifically focused on the relationship between PD-L1expression in ICs and OS. A total of five studies were included in our analysis, and the combined results showed that PD-L1 expression in ICs was positively correlated with OS (HR = 0.73, 95% CI: 0.57-0.94, *p* = 0.013). FEM was used in view of the absence of significant heterogeneity (I^2 ^= 4.5%, *p* = 0.381) (**Figure 4**).

### 3.4 TILs subtypes and prognosis

#### 3.4.1 CD8^+^ T lymphocyte subset

**Figure 5:**
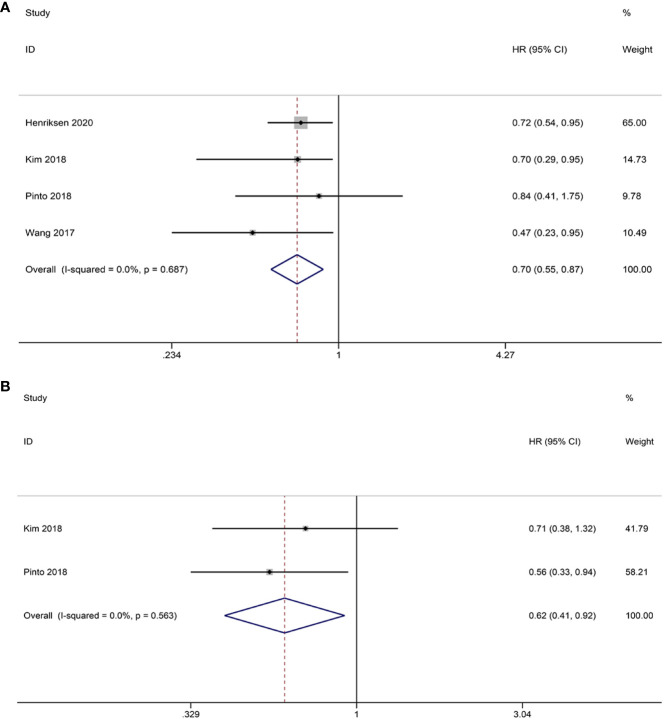
Forest plots of the relationship between CD8^+^ TILs and survival outcomes in HGSOC. **(A)** Forest plots of overall survival (OS); **(B)** Forest plots of progression-free survival (PFS).

Intraepithelial TILs were subjected to analysis in this study. The pooled HRs and 95% CIs indicated that CD8^+^ TILs had a positive impact on OS in patients (HR = 0.70, 95% CI = 0.55-0.87, *p* = 0.002). Given the absence of heterogeneity, FEM was deemed appropriate for analysis (I^2 ^= 0.0%, *p* = 0.687) (**Figure 5A**). Although only two studies provided data on CD8^+^ TILs and PFS, the combined results demonstrated a significant enhancement in patients’ PFS (HR = 0.62; 95% CI= 0.41-0.92, *p* = 0.019). Importantly, no heterogeneity was observed, thus warranting the use of FEM for analysis (I^2 ^= 0.0%, *p* = 0.563) (**Figure 5B**).

#### 3.4.2 PD-1^+^ T lymphocyte subset

**Figure 6:**
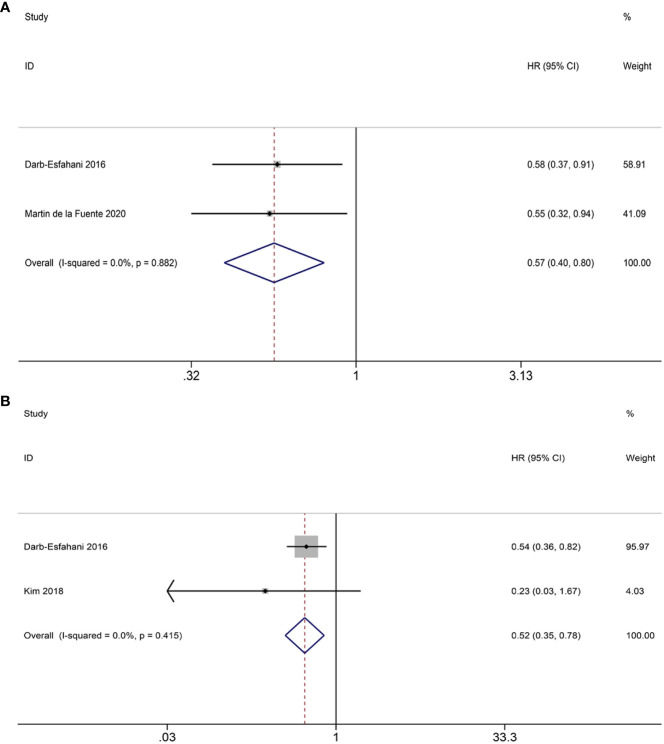
Forest plots of the relationship between PD-1^+^ TILs and survival outcomes in HGSOC. **(A)** Forest plots of overall survival (OS); **(B)** Forest plots of progression-free survival (PFS).

Only two studies have investigated the potential correlation between PD-1^+^ TILs and OS. The aggregated findings imply that PD-1^+^ TILs have the potential to serve as a beneficial prognostic factor for OS (HR = 0.57; 95% CI = 0.40-0.80, *p* = 0.001) (**Figure 6A**). As for PFS, our analysis was limited to two eligible studies. Our results suggest that PD-1^+^ TILs are associated with improved PFS in patients (HR = 0.52; 95% CI = 0.35-0.78, *p* = 0.002) (**Figure 6B**). Heterogeneity was not observed in either the OS (I^2 ^= 0.0%, *p* = 0.882) or PFS (I^2 ^= 0.0%, *p* = 0.415) analyses, therefore, the FEM was utilized.

#### 3.4.3 CD3^+^ T lymphocyte subset

**Figure 7:**
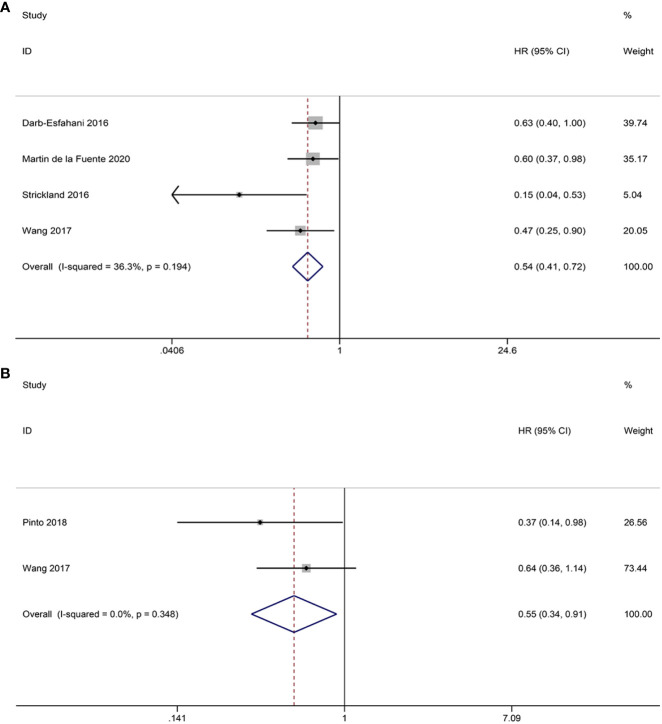
Forest plots of the relationship between CD3^+^, CD4^+^ TILs and overall survival (OS) in HGSOC. **(A)** Forest plot of CD3^+^ TILs; **(B)** Forest plot of CD4^+^ TILs.

Four studies investigated the prognostic significance of CD3^+^ TILs. The pooled analysis revealed the following results: HR = 0.54, 95% CI = 0.41-0.72, *p* = 0.000, suggesting a positive correlation between CD3^+^ TILs and OS. No notable heterogeneity is observed among the studies, thus justifying the use of the FEM (I^2 ^= 36.3%, *p* = 0.194) (**Figure 7A**).

#### 3.4.4 CD4^+^ T lymphocyte subset

Two studies provided data on the relationship between CD4^+^ TILs and OS. The pooled HRs and 95% CIs showed that CD4^+^ TILs were associated with better OS (HR = 0.55, 95% CI = 0.34-0.91, *p* = 0.020). No heterogeneity was observed between studies the two studies, so FEM was used (I^2 ^= 0.0%, *p* = 0.348) (**Figure 7B**).

### 3.5 Publication bias and sensitivity analysis

**Figure 8:**
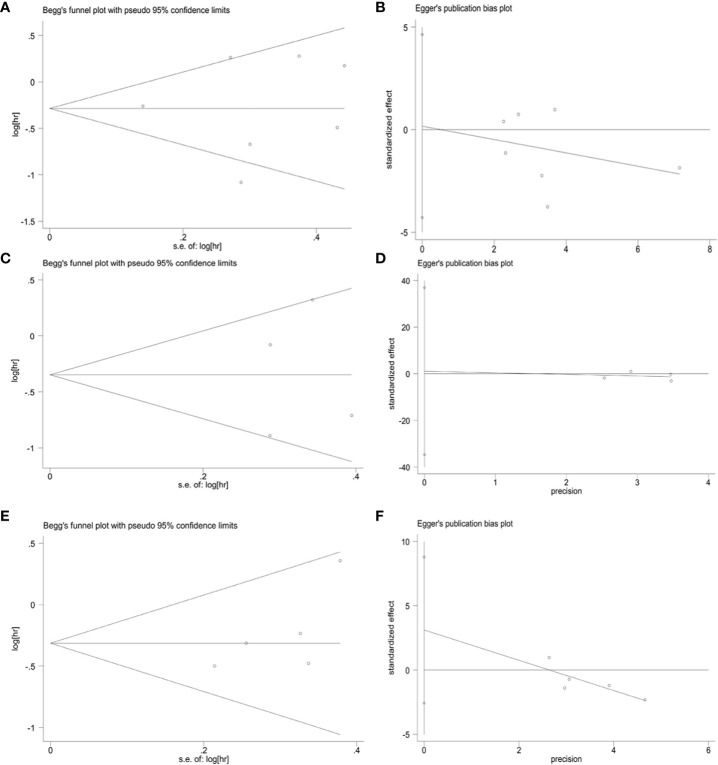
Publication bias. **(A)** Begg’s test for OS in TCs (*p* = 1.000), **(B)** Egger’s test for OS in TCs (*p* = 0.926), **(C)** Begg’s test for PFS in TCs (*p* = 0.734), **(D)** Egger’s test for PFS in TCs (*p* = 0.909), **(E)** Begg’s test for OS in ICs (*p* = 0.308), **(F)** Egger’s test for OS in ICs (*p* = 0.276).

**Figure 9:**
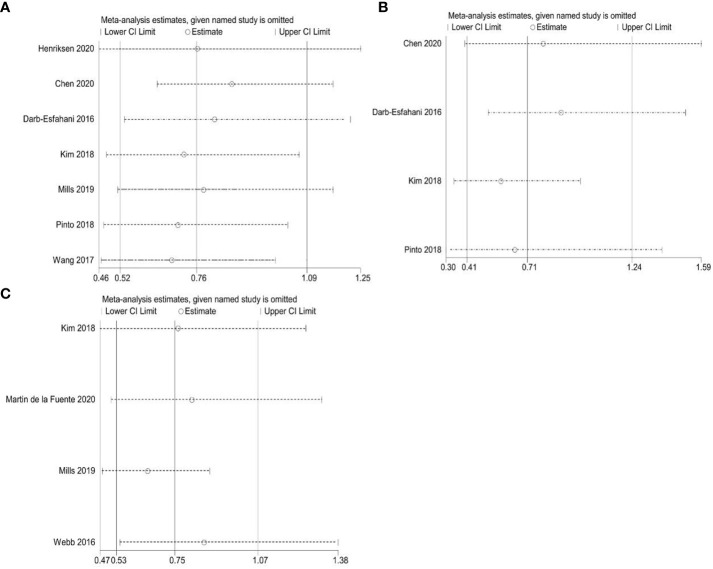
Sensitivity analyses. **(A)** Correlation between PD-L1 expression and overall survival (OS) in TCs; **(B)** Correlation between PD-L1 expression and progression-free survival (PFS) in TCs; **(C)** Correlation between PD-L1 expression and overall survival (OS) in ICs.

To assess publication bias for included studies, we used Begg’s funnel plot and Egger’s linear regression test. For TCs, Begg’s funnel plot presents nearly symmetric features. Begg’s test (OS: *p* = 1.000; PFS: *p* = 0.734) and Egger’s test (OS: *p* = 0.926; PFS: *p* = 0.909) showed no significant publication bias (**Figures 8A–D**). For ICs, publication bias was also not observed (Begg’s test: *p* = 0.308; Egger’s test: *p* = 0.276) (**Figures 8E, F**). In addition, we performed a sensitivity analysis to assess the impact of each study on the overall outcome. Excluding each study individually did not significantly affect the pooled HR (**Figure 9**). This finding highlights the robustness of our pooled results.

## 4 Discussion

Our study demonstrates that PD-L1 expression in TCs is not significantly associated with the prognosis of patients with HGSOC. However, PD-L1 expression derived from ICs is linked to better survival outcomes. These findings indicate that PD-L1 exhibits distinct prognostic values through two different patterns. Importantly, our observation that PD-L1 expression in TCs does not contribute to the development of HGSOC is consistent with previous investigations (30, 31). Accumulating evidence suggests that PD-L1 in TCs does not promote primary tumor formation and progression. However, it may facilitate the metastasis of malignant tumors through the interacting with myeloid cell-derived PD-L1, hindering the infiltration of cytotoxic T cells into the affected area (32). These findings may further support our results, as metastatic lesions were excluded from this study. Numerous studies utilizing mouse models have indicated that PD-L1 expression in ICs is a crucial risk factor for the impairment of T cell-mediated antitumor response (33). However, our results proposed that PD-L1 in ICs correlates with an improved OS of HGSOC patients. These observations are in good agreement with previous research on esophageal cancer (7), which demonstrated that PD-L1 expression in ICs serves as an independent prognostic factor that significantly prolongs patient survival. Furthermore, Kowanetz et al. (34) also reported a positive association between PD-L1 expression in ICs and improved outcomes in patients with non-small cell lung cancer. These data reveal an interesting idea that PD-L1 expression not only plays a positive role in immunosuppression, but also plays a crucial role in endogenous anti-tumor immune responses, leading to inconsistencies in PD-L1 as a prognostic indicator. This inconsistency may not only be related to individual patient differences and tumor heterogeneity, but also be affected by the complex TME.

In fact, PD-L1 expression in the TME is influenced by a variety of interrelated regulatory factors, which makes it dynamic and complex. A variety of ICs exist in the TME, including tumor-associated macrophages, dendritic cells, NK cells, T cells, etc. (35). These ICs and their secreted cytokines play an important role in regulating PD-L1 expression. In general, interferon γ (IFN-γ) produced by activated T cells and NK cells is the main stimulator, which can up-regulate PD-L1 expression by activating the JAK/STAT and PI3K-AKT signaling pathways (36). Recent studies have shown that, in addition to the direct effects on TCs, tumor necrosis factor-α (TNF-α) also induces the expression of PD-L1, thus participating in the escape of TCs from host immune surveillance (37). Other cytokines such as interleukin 6 (IL-6), interleukin 17 (IL-17), and epidermal growth factor (EGF) also directly or indirectly influence PD-L1 behaviors (38, 39). In the setting of pathological conditions, including hypoxia, low nutrients, and stress, an abundant PD-L1 was found in TME (40–42). Barsoum et al. (40) showed that hypoxia-inducing factors can increase the expression of PD-L1 in cancer cells, which, in turn, impedes the effects of cytotoxic T lymphocytes on TCs. At the same time, the expression level of PD-L1 was also interfered by various signaling pathways. Kim et al. (43) found that the activation of PI3K-AKT signaling could lead to an increased expression of PD-L1 in gastric cancer. The administration of PI3K inhibitor (LY294002) significantly decrease the secretion of PD-L1. In addition, Stutvoet et al. (44) confirmed that the activation of EGF-MAPK pathway leads to the upregulation of PD-L1 mRNA and protein in lung adenocarcinoma cells. In conclusion, the expression of PD-L1 in TME represent the result regulated by the combination of multiple exogenous and endogenous factors. This complex regulatory mechanism makes it necessary to take the spatial and temporal dynamic differences into consideration when PD-L1 is served as a prognostic indicator.

It is important to note that different groups arrived at different conclusions about the prognostic value of PD-L1. The regulatory effects of chemotherapy on tumor immune parameters should also be considered. Peng et al. (45) showed that chemotherapy can upregulate PD-L1 expression through the activation of NF−κB signaling pathway in a mouse model of ovarian cancer, leading to local immunosuppression. Meanwhile, Researchers also found that the combination of paclitaxel and anti-PD-L1 drugs significantly increased the survival of murine models with aggressive tumors. Message et al. (46) observed immune cell infiltration and up-regulated PD-L1 expression in the majority of ovarian cancer patients received neoadjuvant chemotherapy (NACT). Consistently, Lee et al. (47) found a NACT-induced increase in PD-L1 expression and TILs density from HGSOC samples. However, these parameters did not result in a survival advantage, which may be partly explained by an increase in the proportion of Foxp3^+^-regulatory T cells. These studies suggest that chemotherapy plays an important and complex role in immune regulation and has a non-negligible impact on PD-L1 expression and prognosis. Due to the limited data provided by the included literature, our study was unable to conduct a comprehensive analysis of the treatment of all patients. In order to better understand the function of PD-L1 and provide tailored treatments, these factors should be taken into account when predicting PD-L1 expression patterns and its impact on prognosis.

TIL refers to a population of lymphocytes that leave the vascular system and infiltrate tumor tissue. Based on molecular markers on the cell surface, such as CD3, CD4, CD8, and PD-1, TILs can be further subdivided into multiple subgroups. The differentiation antigen CD3 is prevalent in mature T cells (48). CD3^+^ TILs represent the level of total T lymphocytes and reflect the status of the host immune function. In keeping with the majority of previous reports (49–51), our findings indicated that the high infiltration of CD3^+^ TILs is significantly associated with better OS of cancer patients and can be served as a reliable biomarker for predicting survival outcomes in HGSOC.

Unlike CD3^+^ TILs, the prognostic value of CD4^+^ TILs has been controversial. Several studies demonstrated that there was no significant correlation between CD4^+^ TILs and prognosis in patients with hepatocellular carcinoma, esophagus cancer, and colorectal cancer (52–54). Conversely, in pancreatic, nasopharyngeal, lung, and bile duct cancers, elevated levels of CD4^+^ TILs have been shown to improve patient survival (50, 55–57). Our findings support the hypothesis that the presence of CD4^+^ TILs in patients with HGSOC can serve as a predictor for an improved survival. The intricate differentiation characteristics of CD4^+^ TILs may contribute to the apparent paradox of behaviors. They can differentiate into multiple T helper (Th) subgroups, thereby effectively enhancing immune responses. Another daughter cell of CD4^+^ TILs is regulatory T cells (Tregs), which are capable of establishing an immunosuppressive microenvironment (58). Because of the acknowledged technical limitations, it is currently unclear if the prognostic significance of CD4^+^ TILs is influenced by the type of cancer, CD4^+^ TILs subgroups, or both. Therefore, further comprehensive investigations and validation of functional subtypes are warranted to explore and confirm the association between CD4^+^ TILs and the prognosis of HGSOC patients.

Another key effector cell involved in the anti-tumor immune response is CD8^+^ TILs, which are able to bind to major histocompatibility complex type I (MHC-I) molecules and directly kill cancer cells through the secretion of granzymes, interferons and other cytokines (59). The favorable prognostic value of CD8^+^ TILs has been extensively studied in various cancers, including head and neck squamous cell carcinoma (49), non-small cell lung cancer (60) and nasopharyngeal carcinoma (50). Consequently, CD8^+^ TILs have emerged as a promising target for novel immunotherapeutic approaches. Our systematic review and meta-analysis provide further support for the notion that increased levels of CD8^+^ TILs significantly improve clinical outcomes in patients with HGSOC. Notably, recent clinical trials utilizing adoptive TILs therapy against melanoma have shown promising results. In these trials, infused CD8^+^ TILs were able to migrate, infiltrate, and eliminate cancer cells, leading to sustained tumor regression in patients (61). Our findings contribute additional evidence supporting the role of CD8^+^ TILs as a reliable prognostic indicator for tumors and their significant potential for augmenting the efficacy of immunotherapy against HGSOC.

The inhibitory molecule PD-1 has attracted much attention as a target for cancer immunomodulation. However, there are still many unveiled areas regarding the relationship between PD-1 expression and patient prognosis. PD-1^+^ TILs numbers increase in direct proportion to disease severity of breast cancer, kidney cancer, nasopharyngeal cancer, and Hodgkin’s lymphoma (62–65). Our results support that PD-1^+^ TILs are more heterogeneous than traditionally thought and may inhibit the progression of HGSOC. Recent studies generally encourage this view that PD-1-expressing TILs convey important anti-tumor effects in follicular lymphoma, head and neck cancer, and colorectal cancer (66–68). These findings have triggered a rethinking of the significance of PD-1^+^ TILs in the battle against cancer. In some cases, PD-1 may serve as an indicator of T cell activation rather than a marker of impaired T cell function (69). Furthermore, given the small amount of literature integrated in this study, a definitive explanation for this discrepancy requires further research in the future to obtain more accurate conclusions.

Our meta-analysis may have some limitations. First, the limited number of included studies may raise concerns about the conclusions. This situation may partly be explained by two reasons. First, the research areas we focused on may be relatively new, resulting in limited available literature resources. Second, to ensure the high quality of the studies, we have developed a strict set of inclusion and exclusion criteria. All literature without reliable methods, high quality, and consistent objects and questions were excluded to improve the scientific rigor and accuracy of our research. Due to the small number of studies included, this study only analyzed the predictive value of intraepithelial TILs and did not investigate stromal TILs in depth. Therefore, it is necessary to further study the infiltration of TILs at different sites to identify possible candidates for immunotherapy. In addition, inconsistencies in the antibodies, assessment methods, and positive thresholds used in the studies may also lead to potential heterogeneity. Future studies should further improve the scoring system and harmonize the cut-off points to obtain more reliable results. Finally, it is important to note that the data extracted from the studies that met the criteria were abstract data rather than individual patient data, which may affect the accuracy of the combined results.

## 5 Conclusions

We found that PD-L1 expression in TCs did not significantly affect long-term survival, but ICs PD-L1 expression was correlated with better OS. Meanwhile, the infiltration of CD8^+^, CD4^+^, CD3^+^, and PD-1^+^ TILs is associated with improved survival. Our results suggest that PD-L1 and TILs may serve as potential biomarkers for personalizing treatment and predicting prognosis in HGSOC patients. However, further well-designed prospective studies are necessary to verify our findings.

## Data availability statement

The original contributions presented in the study are included in the article/**Supplementary Material**. Further inquiries can be directed to the corresponding author.

## Author contributions

Y-MW and WC designed the study. LZ and J-YZ screened the studies and extracted data. The quality of the evidence was assessed using Y-MW, S-YX and WC. Q-MX and J-YZ analyzed and interpreted the data. Y-MW and HD prepared figures and drafted the manuscript. HD contributed to reviewing and editing the manuscript. All authors have approved the final version of the article, including the authorship list. All authors contributed to the article.
