# Supplementary material for: Prognostic role of different PD-L1 expression patterns and tumor-infiltrating lymphocytes in high-grade serous ovarian cancer: a systematic review and meta-analysis
Source: Front Immunol. 2023 Aug 15;14:1234894. doi: 10.3389/fimmu.2023.1234894 (PMC10465691; doi:10.3389/fimmu.2023.1234894)
Supplement: Supplementary file 1 [file DataSheet_1.zip › Frontiers_Supplementary_Material/Supplementary Table 2.docx]

**Supplementary Table 2**. The search strategy for each database.

| **Database** | **Search strategy** |
| --- | --- |
| **PubMed** | **((((((((((((((((((("Ovarian Neoplasms"[Mesh]) OR (Neoplasm, Ovarian[Title/Abstract])) OR (Ovarian Neoplasm[Title/Abstract])) OR (Ovary Neoplasms[Title/Abstract])) OR (Neoplasm, Ovary[Title/Abstract])) OR (Neoplasms, Ovary[Title/Abstract])) OR (Ovary Neoplasm[Title/Abstract])) OR (Neoplasms, Ovarian[Title/Abstract])) OR (Ovary Cancer[Title/Abstract])) OR (Cancer, Ovary[Title/Abstract])) OR (Cancers, Ovary[Title/Abstract])) OR (Ovary Cancers[Title/Abstract])) OR (Ovarian Cancer[Title/Abstract])) OR (Cancer, Ovarian[Title/Abstract])) OR (Cancers, Ovarian[Title/Abstract])) OR (Ovarian Cancers[Title/Abstract])) OR (Cancer of Ovary[Title/Abstract])) OR (Cancer of the Ovary[Title/Abstract])) AND (((((((((((((((((("B7-H1 Antigen"[Mesh]) OR (Antigen, B7-H1[Title/Abstract])) OR (B7 H1 Antigen[Title/Abstract])) OR (Programmed Death Ligand 1[Title/Abstract])) OR (Antigens, CD274[Title/Abstract])) OR (CD274 Antigens[Title/Abstract])) OR (CD274 Antigen[Title/Abstract])) OR (Antigen, CD274[Title/Abstract])) OR (B7-H1 Immune Costimulatory Protein[Title/Abstract])) OR (B7 H1 Immune Costimulatory Protein[Title/Abstract])) OR (B7H1 Immune Costimulatory Protein[Title/Abstract])) OR (PD-L1 Costimulatory Protein[Title/Abstract])) OR (Costimulatory Protein, PD-L1[Title/Abstract])) OR (PD L1 Costimulatory Protein[Title/Abstract])) OR (Programmed Cell Death 1 Ligand 1 Protein[Title/Abstract])) OR (PD-L1 Protein[Title/Abstract])) OR (PD L1 Protein[Title/Abstract])) OR (Programmed Cell Death 1 Ligand 1[Title/Abstract]))) AND (((((((((((((((("Lymphocytes, Tumor-Infiltrating"[Mesh]) OR (Tumor-Infiltrating Lymphocytes[Title/Abstract])) OR (Tumor Infiltrating Lymphocyte[Title/Abstract])) OR (Infiltrating Lymphocyte, Tumor[Title/Abstract])) OR (Infiltrating Lymphocytes, Tumor[Title/Abstract])) OR (Lymphocyte, Tumor Infiltrating[Title/Abstract])) OR (Lymphocytes, Tumor Infiltrating[Title/Abstract])) OR (Tumor Infiltrating Lymphocytes[Title/Abstract])) OR (Tumor-Infiltrating Lymphocyte[Title/Abstract])) OR (Lymphocyte, Tumor-Infiltrating[Title/Abstract])) OR (Tumor-Derived Activated Cells[Title/Abstract])) OR (Activated Cell, Tumor-Derived[Title/Abstract])) OR (Activated Cells, Tumor-Derived[Title/Abstract])) OR (Tumor Derived Activated Cells[Title/Abstract])) OR (Tumor-Derived Activated Cell[Title/Abstract])) OR (Tumor Derived Activated Cell[Title/Abstract]))** |
| **Web of Science** | **(TS=(Ovarian Neoplasms) OR AB=(Neoplasm, Ovarian OR Ovarian Neoplasm OR Ovary Neoplasms OR Neoplasm, Ovary OR Neoplasms, Ovary OR Ovary Neoplasm OR Neoplasms, Ovarian OR Ovary Cancer OR Cancer, Ovary OR Cancers, Ovary OR Ovary Cancers OR Ovarian Cancer OR Cancer, Ovarian OR Cancers, Ovarian OR Ovarian Cancers OR Cancer of Ovary OR Cancer of the Ovary)) AND (TS=(B7-H1 Antigen) OR AB=(Antigen, B7-H1 OR B7 H1 Antigen OR Programmed Death Ligand 1 OR Antigens, CD274 OR CD274 Antigens OR CD274 Antigen OR Antigen, CD274 OR B7-H1 Immune Costimulatory Protein OR B7 H1 Immune Costimulatory Protein OR B7H1 Immune Costimulatory Protein OR PD-L1 Costimulatory Protein OR Costimulatory Protein, PD-L1 OR PD L1 Costimulatory Protein OR Programmed Cell Death 1 Ligand 1 Protein OR PD-L1 Protein OR PD L1 Protein OR Programmed Cell Death 1 Ligand 1)) AND (TS=(Lymphocytes, Tumor-Infiltrating) OR AB=(Tumor-Infiltrating Lymphocytes OR Tumor Infiltrating Lymphocyte OR Infiltrating Lymphocyte, Tumor OR Infiltrating Lymphocytes, Tumor OR Lymphocyte, Tumor Infiltrating OR Lymphocytes, Tumor Infiltrating OR Tumor Infiltrating Lymphocytes OR Tumor-Infiltrating Lymphocyte OR Lymphocyte, Tumor-Infiltrating OR Tumor-Derived Activated Cells OR Activated Cell, Tumor-Derived OR Activated Cells, Tumor-Derived OR Tumor Derived Activated Cells OR Tumor-Derived Activated Cell OR Tumor Derived Activated Cell))** |
| **Cochrane Library** | **#1. MeSH descriptor: [Ovarian Neoplasms] explode all trees** |
|  | **#2. (Neoplasm, Ovarian or Ovarian Neoplasm or Ovary Neoplasms or Neoplasm, Ovary or Neoplasms, Ovary or Ovary Neoplasm or Neoplasms, Ovarian or Ovary Cancer or Cancer, Ovary or Cancers, Ovary or Ovary Cancers or Ovarian Cancer or Cancer, Ovarian or Cancers, Ovarian or Ovarian Cancers or Cancer of Ovary or Cancer of the Ovary):ti,ab,kw (Word variations have been searched)** |
|  | **#3. #1 or #2** |
|  | **#4. MeSH descriptor: [Lymphocytes, Tumor-Infiltrating] explode all trees** |
|  | **#5. (Tumor-Infiltrating Lymphocytes or Tumor Infiltrating Lymphocyte or Infiltrating Lymphocyte, Tumor or Infiltrating Lymphocytes, Tumor or Lymphocyte, Tumor Infiltrating or Lymphocytes, Tumor Infiltrating or Tumor Infiltrating Lymphocytes or Tumor-Infiltrating Lymphocyte or Lymphocyte, Tumor-Infiltrating or Tumor-Derived Activated Cells or Activated Cell, Tumor-Derived or Activated Cells, Tumor-Derived or Tumor Derived Activated Cells or Tumor-Derived Activated Cell or Tumor Derived Activated Cell):ti,ab,kw (Word variations have been searched)** |
|  | **#6. #4 or #5** |
|  | **#7. MeSH descriptor: [B7-H1 Antigen] explode all trees** |
|  | **#8. (B7 H1 Antigen or Programmed Death Ligand 1 or Antigens, CD274 or CD274 Antigens or CD274 Antigen or Antigen, CD274 or B7 H1 Immune Costimulatory Protein or B7H1 Immune Costimulatory Protein or PD L1 Costimulatory Protein or Programmed Cell Death 1 Ligand 1 Protein or PD L1 Protein or Programmed Cell Death 1 Ligand 1):ti,ab,kw (Word variations have been searched)** |
|  | **#9. #7 or #8** |
|  | **#10. #3 and #6 and #9** |
| **Embase** | **#1. 'ovary tumor'/exp** |
|  | **#2. 'neoplasm, ovarian':ab,ti OR 'ovarian neoplasm':ab,ti OR 'ovary neoplasms':ab,ti OR 'neoplasm, ovary':ab,ti OR 'neoplasms, ovary':ab,ti OR 'ovary neoplasm':ab,ti OR 'neoplasms, ovarian':ab,ti OR 'ovary cancer':ab,ti OR**  **'cancer, ovary':ab,ti OR 'cancers, ovary':ab,ti OR 'ovary cancers':ab,ti OR 'ovarian cancer':ab,ti OR 'cancer, ovarian':ab,ti OR 'cancers, ovarian':ab,ti OR 'ovarian cancers':ab,ti OR 'cancer of ovary':ab,ti OR 'cancer of the ovary':ab,ti** |
|  | **#3. #1 OR #2** |
|  | **#4. 'programmed death 1 ligand 1'/exp** |
|  | **#5. 'antigen, b7-h1':ab,ti OR 'b7 h1 antigen':ab,ti OR 'programmed death ligand 1':ab,ti OR 'antigens, cd274':ab,ti OR 'cd274 antigens':ab,ti OR 'cd274 antigen':ab,ti OR 'antigen, cd274':ab,ti OR 'b7-h1 immune costimulatory protein':ab,ti OR 'b7 h1 immune costimulatory protein':ab,ti OR 'b7h1 immune costimulatory protein':ab,ti OR 'pd-l1 costimulatory protein':ab,ti OR 'costimulatory protein, pd-l1':ab,ti OR 'pd l1 costimulatory protein':ab,ti OR 'programmed cell death 1 ligand 1 protein':ab,ti OR 'pd-l1 protein':ab,ti OR 'pd l1 protein':ab,ti OR 'programmed cell death 1 ligand 1':ab,ti** |
|  | **#6. #4 OR #5** |
|  | **#7. 'tumor associated leukocyte'/exp** |
|  | **#8. 'tumor-infiltrating lymphocytes':ab,ti OR 'tumor infiltrating lymphocyte':ab,ti OR 'infiltrating lymphocyte, tumor':ab,ti OR 'infiltrating lymphocytes, tumor':ab,ti OR 'lymphocyte, tumor infiltrating':ab,ti OR 'lymphocytes, tumor infiltrating':ab,ti OR 'tumor infiltrating lymphocytes':ab,ti OR 'tumor-infiltrating lymphocyte':ab,ti OR 'lymphocyte, tumor-infiltrating':ab,ti OR 'tumor-derived activated cells':ab,ti OR 'activated cell, tumor-derived':ab,ti OR 'activated cells, tumor-derived':ab,ti OR 'tumor derived activated cells':ab,ti OR 'tumor-derived activated cell':ab,ti OR 'tumor derived activated cell':ab,ti** |
|  | **#9. #7 OR #8** |
|  | **#10. #3 AND #6 AND #9** |
| **Scopus** | **(( TITLE-ABS-KEY ( ovarian AND neoplasms ) OR TITLE-ABS-KEY ( neoplasm, AND ovarian ) OR TITLE-ABS-KEY ( ovarian AND neoplasm ) OR TITLE-ABS-KEY ( ovary AND neoplasms ) OR TITLE-ABS-KEY ( neoplasm, AND ovary ) OR TITLE-ABS-KEY ( neoplasms, AND ovary ) OR TITLE-ABS-KEY ( ovary AND neoplasm ) OR TITLE-ABS-KEY ( neoplasms, AND ovarian ) OR TITLE-ABS-KEY ( ovary AND cancer ) OR TITLE-ABS-KEY ( cancer, AND ovary ) OR TITLE-ABS-KEY ( cancers, AND ovary ) OR TITLE-ABS-KEY ( ovary AND cancers ) OR TITLE-ABS-KEY ( ovarian AND cancer ) OR TITLE-ABS-KEY ( cancer, AND ovarian ) OR TITLE-ABS-KEY ( cancers, AND ovarian ) OR TITLE-ABS-KEY ( ovarian AND cancers ) OR TITLE-ABS-KEY ( cancer AND of AND ovary ) OR TITLE-ABS-KEY ( cancer AND of AND the AND ovary ) )) AND ((TITLE-ABS-KEY(B7-H1 Antigen) OR TITLE-ABS-KEY(Antigen, B7-H1) OR TITLE-ABS-KEY(B7 H1 Antigen) OR TITLE-ABS-KEY(Programmed Death Ligand 1) OR TITLE-ABS-KEY(Antigens, CD274) OR TITLE-ABS-KEY(CD274 Antigens) OR TITLE-ABS-KEY(CD274 Antigen) OR TITLE-ABS-KEY(Antigen, CD274) OR TITLE-ABS-KEY(B7-H1 Immune Costimulatory Protein) OR TITLE-ABS-KEY(B7 H1 Immune Costimulatory Protein) OR TITLE-ABS-KEY(B7H1 Immune Costimulatory Protein) OR TITLE-ABS-KEY(PD-L1 Costimulatory Protein) OR TITLE-ABS-KEY(Costimulatory Protein, PD-L1) OR TITLE-ABS-KEY(PD L1 Costimulatory Protein) OR TITLE-ABS-KEY(Programmed Cell Death 1 Ligand 1 Protein) OR TITLE-ABS-KEY(PD-L1 Protein) OR TITLE-ABS-KEY(PD L1 Protein) OR TITLE-ABS-KEY(Programmed Cell Death 1 Ligand 1))) AND ((TITLE-ABS-KEY(Lymphocytes, Tumor-Infiltrating) OR TITLE-ABS-KEY(Tumor-Infiltrating Lymphocytes) OR TITLE-ABS-KEY(Tumor Infiltrating Lymphocyte) OR TITLE-ABS-KEY(Infiltrating Lymphocyte, Tumor) OR TITLE-ABS-KEY(Infiltrating Lymphocytes, Tumor) OR TITLE-ABS-KEY(Lymphocyte, Tumor Infiltrating) OR TITLE-ABS-KEY(Lymphocytes, Tumor Infiltrating) OR TITLE-ABS-KEY(Tumor Infiltrating Lymphocytes) OR TITLE-ABS-KEY (Tumor-Infiltrating Lymphocyte) OR TITLE-ABS-KEY(Lymphocyte, Tumor-Infiltrating) OR TITLE-ABS-KEY(Tumor-Derived Activated Cells) OR TITLE-ABS-KEY(Activated Cell, Tumor-Derived) OR TITLE-ABS-KEY(Activated Cells, Tumor-Derived) OR TITLE-ABS-KEY(Tumor Derived Activated Cells) OR TITLE-ABS-KEY(Tumor-Derived Activated Cell) OR TITLE-ABS-KEY(Tumor Derived Activated Cell)))** |
